# Progress in Lactate Metabolism and Its Regulation via Small Molecule Drugs

**DOI:** 10.3390/molecules29235656

**Published:** 2024-11-29

**Authors:** Jin Liu, Feng Zhou, Yang Tang, Linghui Li, Ling Li

**Affiliations:** School of Pharmacy, Hunan University of Chinese Medicine, Changsha 410208, China; 19397862642@163.com (J.L.);

**Keywords:** lactate, small molecule drugs, glycolysis, physiology and diseases

## Abstract

Lactate, once viewed as a byproduct of glycolysis and a metabolic “waste”, is now recognized as an energy-providing substrate and a signaling molecule that modulates cellular functions under pathological conditions. The discovery of histone lactylation in 2019 marked a paradigm shift, with subsequent studies revealing that lactate can undergo lactylation with both histone and non-histone proteins, implicating it in the pathogenesis of various diseases, including cancer, liver fibrosis, sepsis, ischemic stroke, and acute kidney injury. Aberrant lactate metabolism is associated with disease onset, and its levels can predict disease outcomes. Targeting lactate production, transport, and lactylation may offer therapeutic potential for multiple diseases, yet a systematic summary of the small molecules modulating lactate and its metabolism in various diseases is lacking. This review outlines the sources and clearance of lactate, as well as its roles in cancer, liver fibrosis, sepsis, ischemic stroke, myocardial infarction, and acute kidney injury, and summarizes the effects of small molecules on lactate regulation. It aims to provide a reference and direction for future research.

## 1. Introduction

Under conditions of hypoxia or high-intensity exercise, cells undergo a metabolic process wherein glucose is converted to pyruvate, which is then reduced to lactate through the glycolytic pathway. The production of lactate signifies an adaptive cellular response that facilitates the swift acquisition of energy. It is frequently a byproduct of glycolysis and has historically been regarded as a metabolic “waste product”. However, with the progression of scientific research, it has become apparent that lactate plays a much more substantial role in living organisms than was previously assumed. Lactate not only acts as a source of cellular energy but also functions as a signaling molecule with the ability to regulate cellular physiological processes [1]. In 2019, Zhang et al. made notable advancements in the investigation of chemical modifications to histones, which potentially affect gene expression. They successfully predicted and discovered Kla to be a novel histone marker that is responsive to lactate [2]. The discovery of lactylation modification offers a novel perspective on the function of lactate in cellular regulation. However, the excessive accumulation of lactate is also closely associated with the development of several pathological conditions. Elevated lactate levels may be indicative of underlying pathology in the liver, kidneys, heart muscle, and other organs. Under these circumstances, the significant production of lactate can lead to toxicity and dysfunction within the organism. It is encouraging to note that some small molecule drugs are capable of modulating lactate levels. These small molecule regulate lactate production and metabolism through a variety of mechanisms and signaling pathways, thereby providing new avenues for the treatment of diseases related to lactate. To optimize the utilization of lactate as a biomolecule, thorough research is vital to grasp its origins, clearance mechanisms, physiological roles, and connections to diseases. Concurrently, the identification of small molecule drugs that influence lactate levels has led to the introduction of innovative therapeutic approaches and ideas.

## 2. Source of Lactate

**Figure 1:**
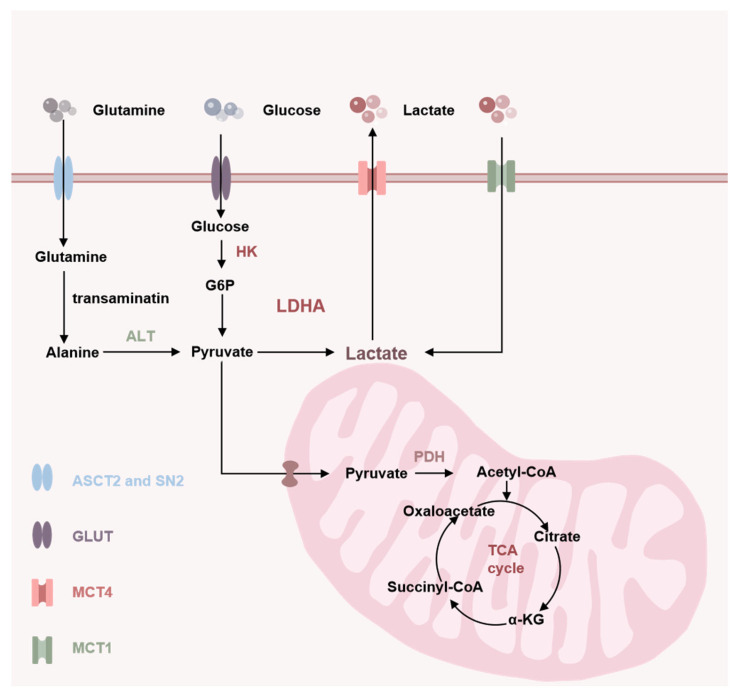
The source of lactate transportation.

In accordance with the prevailing physiological conditions, the foodstuffs consumed by the body are subjected to a series of intricate biochemical processes, ultimately resulting in the conversion of these substances into glucose. As shown in Figure 1, these glucose molecules are transported into the cell via the glucose transporter protein (GLUT) on the cell membrane [3]. Subsequently, glucose is converted to glucose-6-phosphate (G6P) by hexokinase (HK) [4]. G6P is converted to pyruvate (PA) via a series of enzymatic reactions. In the presence of adequate oxygen, pyruvate continues its journey into the mitochondria. In the presence of adequate oxygen, pyruvate is transported into the mitochondria by the mitochondrial pyruvate carrier (MPC) [5], where it is converted to acetyl-CoA by pyruvate dehydrogenase (PDH). This process generates adenosine triphosphate (ATP), providing the energy necessary for cellular functions [6]. However, under hypoxic conditions, pyruvate within the cell is unable to produce energy through the mitochondrial pathway. Instead, it is converted to lactate, which generates ATP through the action of lactate dehydrogenase (LDH) [7]. It is noteworthy that cancer cells, even when exposed to an oxygen-rich environment, tend to adopt the aerobic glycolytic pathway, also known as the Warburg effect, to obtain energy through the production of lactate [8]. Another source of lactate production is glutamine and amino acid catabolism. Glutamylamine and amino acid catabolism represent an additional source of lactate production. When cells utilize glutamyl ammonia and other amino acids, they typically undergo decomposition and transamination reactions, which are accompanied by the production of substantial quantities of alanine. A proportion of alanine is transaminated to pyruvate as a result of the action of alanine aminotransferase (ALT), while pyruvate is catalyzed by LDHA to produce lactate [9].

## 3. Clearance of Lactate

Lactate accumulation occurs when intracellular lactate is not excreted in a timely manner. The accumulated lactate has the potential to cause harm to the human body. For instance, a substantial accumulation of lactate in the serum may precipitate acidosis [10], and the accumulation of lactate in tumor cells has been demonstrated to promote their growth and facilitate immune escape [11]. It is therefore evident that the timely removal of lactate plays a crucial role in maintaining the homeostasis of the human microenvironment. Lactate clearance is primarily accomplished through a multitude of metabolic pathways. Lactate can undergo an irreversible clearance process in the human body via the metabolic enzyme PDH [12] while cells depend on monocarboxylate transporters (MCTs) and exosomes (Exos) to facilitate the efficient removal of lactate. The MCT family comprises 14 members, collectively constituting the solute carrier family 16 (SLC16), these transporters play an integral role in the transport of cellular nutrients and in cellular metabolism [13]. MCT1 is principally accountable for the conveyance of lactate from glycolytic cells, such as tumor cells, to oxidative cells, such as cardiomyocytes and slow muscle fibers, for subsequent oxidative utilization, a process designated the “lactate shuttle”, MCT4 is a high-affinity lactate transporter that exports lactate in a high-lactate microenvironment, thereby maintaining equilibrium between intracellular and extracellular lactate levels [14]. Exosomes are a type of cystic vesicle secreted by active cells. They are capable of transporting macromolecular signals, including lipids, proteins, and genetic materials, from the interior of the cell to the extracellular space [15]. Recent studies have revealed that lactate can act not only as a metabolite but also as a signaling molecule to stimulate cells to produce exosomes. For example, lactate promotes the lactylation of HMGB1 through a p300/CBP-dependent mechanism, and the lactylation of HMGB1 can then be transported outside the cell via exosomes produced by lactate-stimulated cells [16]. Consequently, the clearance of lactate is accomplished indirectly. Lactate clearance has the potential to restore the normal physiological function of cells and maintain the normal metabolic processes and oxidation of cells.

## 4. The Role of Lactate in Biological Processes

### 4.1. Regulation of Energy

**Figure 2:**
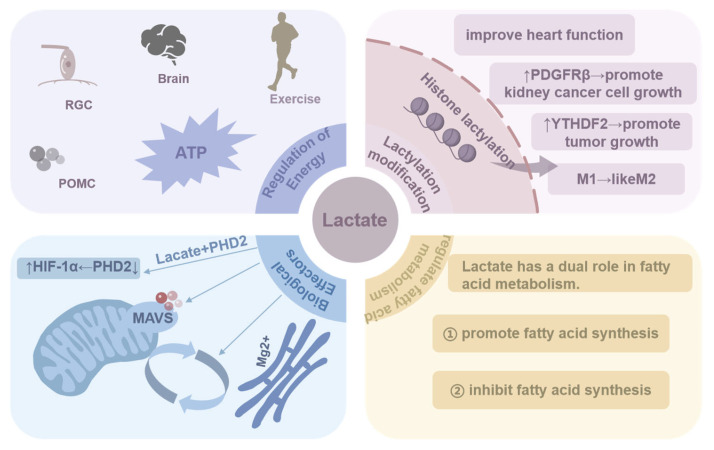
The physiological functions involving lactate.

We describe The Role of Lactate in Biological Processes as shown in Table 1 and Figure 2. Lactate plays a significant role in the physiological processes of the human body, both under normal physiological conditions and in the context of pathological processes. In normal physiological activities, the concentration of lactate in the human bloodstream increases in response to physical exertion [17]. The necessity of rapid energy acquisition during exercise is a primary factor in this phenomenon. While the tricarboxylic acid cycle is capable of providing a substantial amount of energy, its intricate and time-consuming process renders it inadequate for the demands of exercise. In contrast, the process of glycolysis is more straightforward and occurs more rapidly. The production of lactate is a crucial aspect of the glycolysis pathway, enabling the body to meet its energy demands during exercise [18]. Moreover, lactate has been identified as the primary energy source for specific cells, including retinal ganglion cells (RGCs) [19]. The data indicate that these cells are more likely to utilize lactate as an energy source than glucose, suggesting that lactate plays a unique role in energy metabolism in specific cellular contexts. It has been evidenced that lactate enhances cerebral energy provision and facilitates ATP accumulation under normoglycemic circumstances [20]. This finding offers new insights into our understanding of energy metabolism in the brain. The observed dysregulation of energy metabolism in the precursor melanocortin (POMC) cells induced by MCT inhibitors suggests that these cells may rely primarily on lactate as an energy source [21]. Furthermore, the study of live guinea pig ventricular cardiomyocytes confirmed that lactate activates cardiac ATP-sensitive potassium channels, thereby affecting the electrophysiological characteristics of cardiomyocytes [22]. The aforementioned studies indicate that certain cells and tissues may utilize lactate as an alternative source of energy, instead of glucose. Lactate is essential to human physiological activities. In addition to serving as an energy source for cells during hypoxia, it fulfills distinct functions in various cells and tissues. It serves as the primary source of rapid energy during exercise and is the preferred energy source for select cells, such as retinal ganglion cells. Lactate has been shown to enhance the energy supply to the brain and to influence the electrophysiological properties of cardiac muscle cells. 

### 4.2. Lactylation Modification

Protein modification, also referred to as protein post-translational modification (PTM), entails a series of biochemical reactions that alter the chemical structure of the protein molecule subsequent to mRNA translation [23]. Such alterations have the potential to impact a number of protein characteristics, including structure, stability, and activity [23]. The function of proteins is regulated by cells through a number of different processes, including phosphorylation [24], ubiquitination [25], methylation [26], and acetylation [27]. A novel protein modification, histone lactylation, has recently been identified. In 2019, Zhang D et al. [2] employed high-performance liquid chromatography (HPLC) coupled with tandem mass spectrometry (MS/MS) to demonstrate that lactate can be incorporated into histone lysine residues, thereby conferring M2-like functions in M1-type macrophages at later stages. These findings indicate that histone lactylation may serve as a lactate-based temporal regulator, orchestrating the transition of macrophages from an inflammatory to a homeostatic phenotype. Furthermore, PI3K B cell adapters (BCAP) have been demonstrated to facilitate the transition of macrophages from a pro-inflammatory phenotype to a homeostatic phenotype [28]. Studies have shown that a loss of BCAP results in a reduction in aerobic glycolysis, a decrease in lactate production, and an impairment of histone lactylation, which, in turn, impairs the repair and expression of tissue genes [29]. These findings indicate that BCAP, when modified via histone lactylation, may facilitate the transition of pro-inflammatory macrophages to reparative macrophages [29]. Subsequent to histone lactylation, it may facilitate the expression of m6A-modified protein YTHDF2, thus contributing to the promotion of tumorigenesis [30]. Concurrently, histone lactylation is concentrated in the promoter regions of glycolytic genes, which activate transcription and enhance glycolytic activity [31]. In renal cell carcinoma, histone lactylation levels are elevated, which facilitates the transcription of platelet-derived growth factor receptor β (PDGFRβ) and stimulates the proliferation of renal cell carcinoma [32]. In studies examining sepsis, a positive correlation was observed between the level of histone lactylation and the expression of arginase-1 (Arg1) [33]. The literature indicates that histone lactylation may facilitate the transcription of repair genes, regulate the anti-inflammatory and pro-angiogenic effects of monocytes and macrophages, and thereby contribute to the repair of the microenvironment and the improvement in cardiac function following myocardial infarction [34]. 

Protein modification refers to the chemical alterations to proteins that occur as a result of biochemical reactions subsequent to mRNA translation. These modifications impact the properties of the proteins in question. The recently identified protein lactate modification has been demonstrated to play a pivotal role in a multitude of biological processes, including the regulation of macrophage function, the modulation of glycolytic activity, and the promotion of tumorigenesis. Furthermore, histone lactylation has been linked to the transcription of repair genes and enhanced cardiac function, underscoring the intricate regulatory processes involved. 

### 4.3. Biological Effectors

As research progresses, it becomes increasingly evident that lactate plays a multifaceted and pivotal role in biological systems. In addition to its role as a pivotal enzyme in cellular energy metabolism, it also functions as a signaling molecule, regulating various physiological cellular functions. The study by Xu Aiming et al. [35] revealed the mechanism of lactate accumulation in obese mice and its effect on the inflammatory response of macrophages in adipose tissue. They found that the expression of glycolytic genes was increased in obese mice, while the expression of tricarboxylic acid cycle (TCA) genes was reduced, leading to lactate accumulation. Macrophages have a strong lactate uptake capacity, which allows for lactate from fat to enter adipose tissue macrophages (ATM), thereby promoting ATM polarization and the production of pro-inflammatory factors. Furthermore, lactate can directly bind to prolyl hydroxylase 2 (PHD2) and inhibit its activity, while promoting the protein stability of hypoxia-inducible factor 1α (HIF-1α), thereby aggravating inflammation. This suggests that lactate secreted by adipocytes can act as a “metabolic signaling molecule” to regulate the inflammatory response of macrophages in adipose tissue. In addition, lactate can bind to the transmembrane domain of mitochondrial antiviral signaling proteins (MAVS) and prevent MAVS aggregation, thereby inhibiting pattern recognition receptor (RLR) signaling mediated by glycolysis [36]. The results of this study confirm that lactate directly inhibits RLR signaling and identifies MAVS as a lactate sensor. In addition, lactate is also involved in the regulation of intracellular Mg^2+^ transport. Mg^2+^ transport plays an important role in the regulation of life energy. Lactate can regulate the transfer of Mg^2+^ between the endoplasmic reticulum and mitochondria, act as an intracellular Mg^2+^ kinetic agonist activation signal, and link the mitochondrial Mg^2+^ transport with the main metabolic feedback loop and mitochondrial bioenergy metabolism [37]. The aforementioned studies suggest that lactate, serving as a crucial signaling molecule, exerts a significant influence on the regulation of diverse physiological and pathological processes. Lipids, as a class of highly complex biomolecules within organisms, serve a function that extends far beyond the fundamental structure of biological membranes [38]. 

### 4.4. Regulation of Fatty Acid Metabolism

Lipids are integral to several fundamental biological processes, including cell signaling, energy metabolism, and a variety of other biological functions [39]. In recent years, as research has progressed, the relationship between lactate and lipid metabolism has been elucidated in greater detail. A series of biochemical reactions was observed in rat muscle, whereby lactate is converted to glycerol. The subsequent production of fatty acids is promoted by glycerol, thereby regulating lipid metabolism [40]. Furthermore, lactate secreted by prostate cancer fibroblasts (CAFs) has been demonstrated to regulate intracellular lipid accumulation in lipid droplets (LDs). Lactate regulates the expression of lipid metabolism genes in prostate cancer cells by providing acetyl groups for histone acetylation [41]. This mechanism elucidates the pivotal function of lactate in cancer cell metabolism. In neurons, lactate also fulfills an essential regulatory function. Neurons with higher levels of reactive oxygen species (ROS) showed increased lactate production. Lactate is transported from glial cells to neurons by MCT, where it is converted to phosphatidic acid (PA) and acetyl-CoA [42]. These substances provide the required substrates for fatty acid synthesis, which in turn regulates the adipogenic process. The is a close relationship between lactate and lipid metabolism. Lactate is not only involved in the production of fatty acids and the regulation of fat metabolism but also affects lipid metabolism by affecting the accumulation of lipid droplets and regulating gene expression. Additionally, lactate has been shown to inhibit the catabolism of fatty acids. Specifically, studies conducted on athletes demonstrated a negative correlation between circulating lactate concentrations in the blood during exercise and the level of fatty acid oxidation [43]. Furthermore, a novel compound, LaKe, designed as a simple ester for the dual oral delivery of lactate and β-hydroxybutyrate (BHB), was shown to significantly elevate the plasma levels of both metabolites in rats following oral administration [44]. Concurrently, the ingestion of LaKe led to a decrease in plasma free fatty acid (FFA) levels and an increase in the concentration of the anorexigenic peptide hormone N-L-lactoyl-phenylalanine (Lac-Phe). The inhibitory effect of lactate on fatty acid oxidation may be mediated through an increase in the intracellular NADH/NAD+ ratio, thereby suppressing the β-oxidation process of fatty acids [45,46]. It is noteworthy that the impact of lactate on fatty acid oxidation is multifaceted, and these effects may vary under different physiological and pathological conditions, indicating a complex role for lactate in the regulation of energy metabolism.

**Table 1:** The role of lactate in biological processes.

Biological Processes	Characteristics	Refs.
Regulation of energy	Lactate serves as a fuel for anaerobic exercise; it is an energy source for certain cells (such as PGC and POMC) and also supplies energy to the brain.	[17,18,19,20,21,22]
Lactylation modification	Lactate provides the lactyl group, causing histones to undergo lactylation and promoting a shift from a macrophage inflammatory phenotype to a reparative phenotype. Histone lactylation, which alters gene expression, promotes the expression of multiple proteins (e.g., YTHDF2 and PDGFRβ), which in turn promotes tumor growth.	[2,28,29,30,31,32,33,34]
Biological effector	Lactate acts as a biological effector that contributes to ATM polarization and promotes the production of inflammatory factors; it binds to PDH, inhibits PDH activity, and stabilizes HIF-1α, exacerbating inflammation; it alsobinds to the transmembrane structural domains of the MAVS, promoting glycolysis, and regulates Mg+ transport.	[35,36,37,38]
Regulation of fatty acid metabolism	Lactate plays a dual role in the regulation of fatty acids. On the one hand, lactate is converted to glycerol, which promotes fatty acid production, and it is also converted to PA and acetyl- CoA to regulate fatty acid metabolism. On the other hand, lactate increases the NADH/NAD+ ratio to inhibit the β-oxidation of fatty acids, thus inhibiting fatty acid production.	[40,41,42,43,44,45,46]

## 5. Increased Lactate in the Blood: A New Therapy for Disease

Lactate concentration, serving as a biomarker of disease severity, is frequently elevated in conditions such as acute heart failure (AHF) and sepsis. Traditionally, hyperlactatemia has been associated with poor prognosis; however, recent studies suggest that moderate increases in blood lactate levels may have potential therapeutic benefits for various pathological states, including heart failure [47], cardiogenic shock [48], and brain injury [49]. In a study utilizing a heart failure model in female Danish Landrace pigs, a randomized, double-blind trial compared the effects of a 5 mL/kg/h sodium lactate solution to an isomolar control solution on cardiac function. The pigs treated with sodium lactate demonstrated an increase in cardiac output, an elevated heart rate, and reduced vascular afterload, indicating the positive impact of sodium lactate on cardiac function [47]. Furthermore, a clinical trial involving 40 patients with acute heart failure randomly assigned them to two groups. The intervention group received a 3 mL/kg bolus of half-molar sodium lactate over 15 min, followed by a continuous infusion of 1 mL/kg/h for 24 h, while the control group received only a 3 mL/kg bolus of Hartmann’s solution without continuous infusion. The primary outcome measure was cardiac output (CO), assessed via echocardiography at 24 h, with right ventricular contractility as a secondary outcome. The results showed an increase in CO and heart rate in the intervention group [48]. In the context of traumatic brain injury (TBI), the modulation of lactate levels is also considered to have potential therapeutic effects. TBI can lead to brain edema, microcirculatory disturbances, and oxidative metabolic dysfunction. Treatment with a 3 h hypertonic sodium lactate solution (HSL) was shown to reverse mitochondrial dysfunction induced by TBI and improve brain oxygenation and perfusion, reducing brain edema [49]. Recently, a novel compound named Lake was developed, which, when orally administered to rats, increases plasma lactate levels in a dose-dependent manner, while decreasing plasma free fatty acid (FFA) levels and increasing the levels of anorexigenic peptide hormone N-L-lactoyl-phenylalanine (Lac-Phe) [44]. In summary, lactate, once solely considered a marker of poor prognosis, is now recognized for its potential therapeutic benefits in conditions like acute heart failure, cardiogenic shock, and brain injury, with studies demonstrating its capacity to improve cardiac function, ameliorate brain injury, and modulate metabolic responses in a variety of clinical and preclinical settings.

## 6. Lactate: Bridging Disease and Metabolic Abnormalities

**Figure 3:**
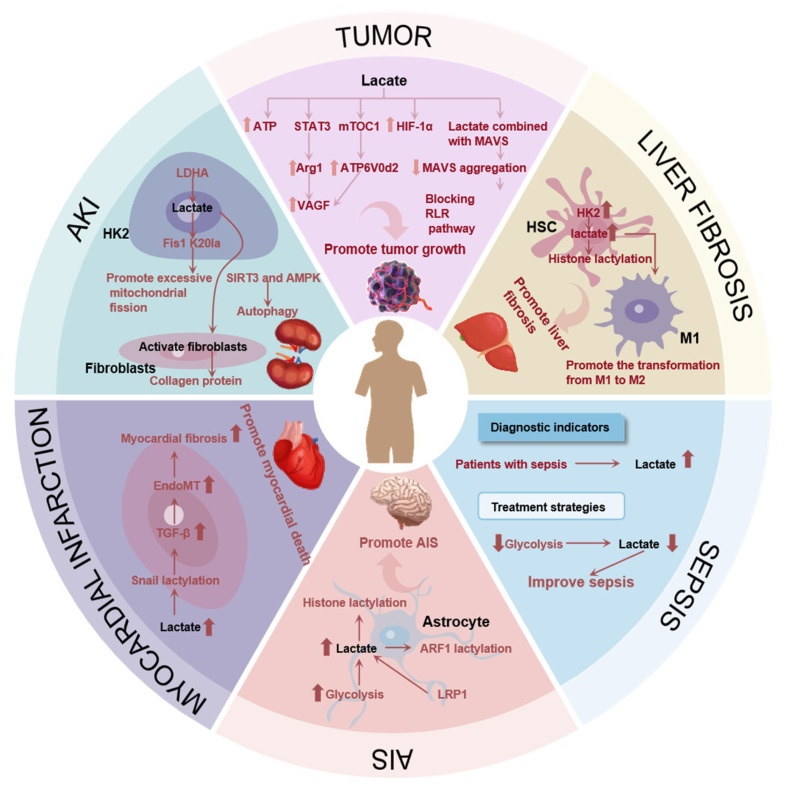
The impact of lactate on disease.

Although the aforementioned statements suggest that appropriately increasing blood lactate levels may improve outcomes in cardiovascular disease or brain injuries, in most cases, elevated lactate within the body can serve as an important indicator for assessing diseases. Lactate plays a significant role in inflammation, immune responses, and signal transduction, affecting the onset of various diseases. For instance, lactate can aid tumor cells in evading immune surveillance. Increased lactate in the liver induces the transformation of macrophages from the M1 phenotype to the M2 phenotype, exacerbating liver fibrosis. Moreover, elevated lactate levels lead to lactylation modifications, altering gene expression and further intensifying the disease. Lactate and LDH can serve as predictive factors for a multitude of diseases; thus, lactate is not only a prognostic clinical marker for various diseases but also a target for the study of disease mechanisms and potential therapeutic strategies. Next, we describe the role of lactate in different diseases, as shown in Figure 3.

### 6.1. Lactate’s Role in Tumor

In 2020, it was estimated that there were 19.3 million new cases of cancer and nearly 10 million cancer-related deaths worldwide [50]. The microenvironment of a tumor is a complex and distinctive ecosystem, markedly distinguished by hypoxia. The substantial reproduction and expansion of tumor cells frequently exceed the capacity of the oxygen supply to meet their metabolic demands, resulting in the formation of a hypoxic microenvironment [51]. This hypoxia state will result in further disruptions to the tumor microenvironment, which will, in turn, affect tumor growth, metastasis, and invasion. In the context of hypoxic conditions, there is the potential for significant alterations to occur in the metabolic pathways of tumor cells [52]. Normally, cells undergo aerobic oxidation through the tricarboxylic acid (TCA) cycle in the presence of sufficient oxygen, thereby generating a substantial amount of energy [9]. However, in an environment with low oxygen levels, the TCA cycle is suppressed, prompting tumor cells to rely on glycolysis for energy production [53]. This metabolic shift can result in the production of substantial quantities of lactate, which serves to further exacerbate the acidification of the tumor microenvironment. Lactate plays a significant role in the tumor microenvironment. In addition to providing energy for tumor cells, lactate plays a role in regulating the growth and proliferation of tumor cells. For instance, in many cancers, including lung, liver, and prostate cancers, the level of lactate is significantly correlated with tumor growth and prognosis [54,55]. In addition, lactate can also affect tumor growth by affecting signaling pathways and immune cell activity in the tumor microenvironment. For example, tumor-derived lactate induces macrophages to convert to M2 by stimulating STAT3 to activate endothelial growth factor (VEGF) and arginine (Arg-1), which further promote tumor development [56]. HIF-1α is a further crucial regulator of the tumor hypoxic microenvironment [57]. In hypoxic conditions, the activation of HIF-α subsequently activates a series of HIF target genes, thereby promoting tumor growth and metastasis [58,59]. Lactate has been demonstrated to reduce intracellular levels of cyclic adenosine monophosphate (cAMP) and the activation of protein kinase A (PKA) through the action of the G protein-coupled receptor 81 (GPR81). Subsequently, the PKA-mediated ubiquitination of HIF-1α results in the inhibition of degradation, thereby causing HIF-1α protein accumulation. Subsequently, HIF-1α induces the transcription of Rab27a, which promotes the release of sEVs; in turn, this promotes tumor growth [60]. Moreover, lactate can also affect the immune surveillance of tumor cells by interacting with MAVS adaptor proteins [61]. Lactate can directly bind to the transmembrane domain of MAVS, thereby inhibiting the aggregation of MAVS and blocking RLR signaling. This enables tumor cells to evade the monitoring and clearance of the immune system [62]. In an anaerobic environment, lactate produced by tumor cells inhibits the expression of the macrophage-specific vacuolar ATPase subunit ATP6V0d2 by stimulating the microtubule-targeting chimera 1 (mTOC1) pathway; this results in an elevation in the HIF-2α-mediated production of VEGF in macrophages, which subsequently display tumor-cell growth-promoting characteristics [63].

The tumor microenvironment is a complex and distinctive entity, distinguished by hypoxia, which instigate alterations in the metabolic pathways of tumor cells, predominantly glycolysis, resulting in the generation of a substantial quantity of lactate and a further exacerbation of the acidification of the microenvironment. Lactate serves not only as an energy source for tumor cells but also as a regulator of their growth and proliferation. Furthermore, it plays a role in tumor development by affecting the signaling pathways and immune cell activity, including through inducing macrophages to transform into the M2 type. HIF is also a crucial regulator, influencing metabolic processes, oxygen delivery, and other biological functions by regulating the expression of hypoxia response genes. Moreover, lactate can interact with MAVS adaptor proteins, thereby affecting tumor cell immune surveillance and enabling tumor cells to evade immune system clearance. In an anaerobic environment, lactate also inhibits the expression of specific genes in macrophages by stimulating specific pathways, thereby promoting the growth of tumor cells.

### 6.2. Lactate’s Role in Liver Fibrosis

The occurrence of liver fibrosis can be regarded as an excessive repair response of the liver to injury. This manifests as the abnormal proliferation and deposition of the extracellular matrix in the liver tissue, the formation of scar tissue, and ultimately, abnormal liver structure or function [64]. This pathological process involves multiple cell types and complex molecular mechanisms [65]. Among the cells involved in the process of liver fibrosis, hepatic stellate cells (HSCs) are of particular importance [66]. In the resting state, HSCs undergo activation and transform into cells with anti-inflammatory and pro-fibrotic characteristics when subjected to disturbances caused by pathogenic factors, such as damaged epithelial cells and immune or metabolic dysregulation [67]. The activation process is accompanied by a significant expenditure of energy, primarily provided by aerobic glycolysis, which results in an increase in the production of lactate [68,69]. In inactivated HSC, HK2 will undergo lactate modification, and in HK2 knockout HSC, the activation of HSC will be inhibited [70]. This further substantiates the impact of lactate on liver fibrosis. Moreover, macrophages within the liver contribute significantly to the development of liver fibrosis. Macrophages are capable of undergoing polarization into either the M1 or M2 phenotype in response to disparate internal environmental stimuli. Upon stimulation by lipopolysaccharide or interferon-γ, M1 macrophages release a substantial number of inflammatory factors, thereby contributing to the immune response against pathogens and facilitating the transition of metabolism to the aerobic glycolysis pathway, which results in the production of lactate [71,72]. In contrast, M2 macrophages produce anti-inflammatory factors and promote the fibrotic process [73]. Studies have shown that the accumulation of lactate in activated HSCs can promote the transformation of macrophages from M1 to M2, thereby further promoting the progression of liver fibrosis [74]. 

Liver fibrosis is defined as an excessive repair response of the liver to injury, which is characterized by the abnormal proliferation and deposition of the extracellular matrix. A multitude of cell types and molecular mechanisms are implicated in this process, with HSCs and macrophages occupying a pivotal position. HSCs are activated when exposed to pathogenic factors and undergo a transformation into cells with anti-inflammatory and pro-fibrotic characteristics, accompanied by increased lactate production. Macrophages can be classified into two distinct polarization states: M1 and M2. The M1 phenotype is anti-inflammatory, whereas the M2 phenotype is pro-fibrotic. The accumulation of lactate has been demonstrated to facilitate the transformation of macrophages from the M1 to the M2 phenotype, which in turn serves to accelerate the progression of liver fibrosis. Therefore, coordinating the interactions between these cells could help to reverse the process of liver fibrosis and restore the normal structure and function of the liver.

### 6.3. Lactate’s Role in Sepsis

The mechanism of sepsis is a complex and multifaceted process, which involves a variety of factors, such as immune response triggered by infectious agents, immunosuppression, coagulation dysfunction, and organ dysfunction [75]. The interaction of these factors leads to the appearance of systemic inflammatory response syndrome, which in turn may trigger sepsis. In the context of sepsis development, alterations in lactate levels possess diagnostic value. The level of lactate is closely related to the prognosis of patients with sepsis [76]. The higher the lactate level, the more severe the tissue hypoperfusion and the worse the patient’s prognosis [77]. Accordingly, lactate levels can be monitored to ascertain the severity and prognosis of sepsis patients. Moreover, lactate modification has emerged as a promising marker for the treatment and diagnosis of sepsis in recent years [78]. Inhibition of the isoenzyme PFKFB3, which is involved in the glycolytic pathway, has been demonstrated to reduce lactate production, thereby improving the symptoms of sepsis [79]. These findings indicate that the regulation of the glycolytic pathway may represent a novel strategy for the treatment of sepsis. 

### 6.4. Lactate’s Role in Ischemic Stroke

Stroke represents the second leading cause of disability and mortality at a global scale, with ischemic stroke (AIS) accounting for approximately 87% of cases [80]. AIS is typified by high rates of recurrence, mortality, and disability [81]. LDH, functioning as a lactate convertase, has been employed as a biomarker for a number of pathological conditions [82]. Some scholars have identified the presence of LDH in the serum of AIS patients, noting an elevated LDH content in these individuals. This observation may serve as a potential biomarker for the diagnosis of AIS in the future [83]. In the context of cerebral ischemia, astrocytes rely on the glycolytic process as their primary source of energy. This is accompanied by the inhibition of OXPHOS and disruption of mitochondrial function, which collectively result in an elevated lactate content within the cerebrospinal fluid [84]. An elevated lactate level will serve to exacerbate the effects of an ischemic stroke [85]. Previous studies have shown that reducing lactate content by inhibiting LDHA or glycolysis can significantly improve brain damage in mice with ischemic stroke. One study used a pan-antibody for lactacitization to detect protein lactacitization in astrocytes and found that protein lactacitization was upregulated in an ischemic stroke model [85], but the study did not further indicate which protein was lactated. Low-density lipoprotein receptor (LDLR)-related protein-1 (LRP1) is a multifunctional transmembrane protein [86]; LRP1 has been reported to be associated with AIS [87]. In a mouse model of ischemic stroke, the inhibition of LRP1 in astrocytes impeded the transfer of mitochondria to neurons, thereby exacerbating ischemic stroke [88]. This could provide further clarification regarding the inhibitory effect of LRP1 in astrocytes on lactate production and the reduction in ADP-ribosylation factor 1 (ARF1) lactylation, as well as the promotion of astrocyte mitochondrial transfer to neurons and the alleviation of ischemic stroke [88]. In SAI, there is an increase in lactate content in both the serum and cerebrospinal fluid. These elevated lactate levels lead to the protein becoming a protein lactate, which contributes to ischemic stroke.

### 6.5. Lactate’s Role in Myocardial Infarction

Myocardial infarction (MI) is a pathological condition resulting from prolonged ischemia of the heart or coronary arteries [89]. This is typically accompanied by hypoxia and an imbalance in energy metabolism within the cardiomyocytes [90]. In a hypoxic microenvironment, the mitochondrial oxidative phosphorylation of cardiomyocytes is reduced while glycolysis is enhanced, which ultimately results in elevated lactate levels [91]. Clinical studies have corroborated the notion that the concentration of lactate in patients who have experienced an MI will increase. The measurement of serum lactate levels is of paramount importance for the prognostic evaluation of patients who have suffered a myocardial infarction [92,93]. As early as 1991, it was proposed that peripheral blood lactate levels could serve as a diagnostic indicator for myocardial infarction [94]. In addition, myocardial infarction is often accompanied by cardiac fibrosis in patients [95]. Although early cardiac fibrosis has an inhibitory effect on the rupture of infarcted cardiac tissue at the time of myocardial infarction, persistent cardiac fibrosis is a significant contributing factor to the development of heart failure [96]. Endothelial-to-mesenchymal transition (EndoMT) plays an important role in cardiac fibrosis. MI results in myocardial cell necrosis and interstitial fibrosis, whereas EndoMT can exacerbate cardiac fibroblast generation and collagen deposition, thereby further aggravating cardiac fibrosis [97]. Transforming growth factor beta (TGF-β) plays a pivotal role in the activation of EndoMT. It stimulates the phosphorylation of Smad2/3, thereby promoting the expression of EndoMT transcription factors, including Snail1 [98]. Some researchers have discovered that inhibiting lactate production using 2-DG can enhance the mitigation of myocardial infarction-induced EndoMT and cardiac fibrosis, thereby alleviating the symptoms of myocardial infarction. However, the addition of lactate has been observed to exacerbate both EndoMT and myocardial infarction. Lactate has been demonstrated to upregulate TGF-β expression by promoting the lactoacylation of Snail1, a transcription factor of TGF-β, and enhancing its nuclear translocation and binding to the TGF-β promoter [99]. In conclusion, lactate levels are increased in patients with myocardial infarction, and elevated lactate upregulates TGF-β by promoting the lactoacylation of Snail1, inducing EndoMT and cardiac fibrosis and thereby exacerbating myocardial infarction.

### 6.6. Lactate’s Role in Acute Kidney Injury

Acute kidney injury (AKI) is a clinical syndrome characterized by a rapid decline in kidney function, which may be caused by a variety of factors [100]. It is estimated to cause a mortality rate as high as 20–50% worldwide [101]. Clinical studies have shown that the serum lactate content of AKI patients is significantly increased, and the increase in lactate will further aggravate the course of the disease [102]. In a mouse model of AKI, the addition of exogenous lactate aggravated the histopathological damage and apoptosis of the kidney [103]. Mitochondrial fission 1 protein (Fis1), which serves as an adaptor protein on the mitochondrial outer membrane, interacts with the fission execution gene dynamin-related protein 1 (DRP1) to facilitate mitochondrial fission [104]. In vitro and in vivo experiments have evidenced that the incorporation of lactate can facilitate the lactoacylation of Fis1 lysine 20 (Fis1 K20la), which in turn induces excessive mitochondrial fission and dysfunction, thereby exacerbating kidney injury [103]. In addition, autophagy plays a protective role in AKI. When AKI occurs, autophagy is activated in renal tubular epithelial cells. Blocking autophagy can further aggravate kidney injury, and inducing autophagy can help alleviate kidney injury [105]. The mRNA levels of lactate and glycolysis-related proteins, including PKM2 and LDHA, were elevated in HK2 cells that were induced by LPS. Conversely, the autophagy process was enhanced when the glycolytic inhibitor 2-DG was employed. Subsequent research demonstrated that lactate impeded the induction of autophagy by suppressing the autophagy regulators SIRT3 and AMPK [106]. Additionally, fibroblasts are a significant contributor to the pathogenesis of AKI. The proliferation of fibroblasts is increased in the renal interstitium of numerous AKI models, and the activation of fibroblasts plays a pivotal role in the process of renal tubular injury, repair, and recovery following AKI [107,108,109]. In AKI, glucose metabolism in renal tubular epithelial cells is dominated by glycolysis, producing large amounts of lactate. A lactate-rich microenvironment can lead to the activation of fibroblasts and promote collagen deposition, which has a reparative effect in the early stage of AKI; however, excessive collagen deposition can lead to renal fibrosis [110]. Thus, the inhibition of lactate production inhibits fibroblast activation and collagen deposition in a model of AKI, potentially contributing to the reduction in kidney injury.

## 7. Small Molecule Drugs for Regulating Lactate Levels

Lactate, a principal product of the glycolytic pathway, has the potential to elicit a multitude of pathological responses when its accumulation is excessive, include promoting tumor growth, accelerating the process of liver fibrosis, and promoting the development of diseases such as sepsis and ischemic stroke. The final product of this metabolic pathway is the regulation of lactate production via key enzymes involved in glycolysis, including HK-2, and LDHA. In hypoxic conditions, HIF-1α upregulates the expression of glycolysis-related genes (glucose transporters, HK, etc.), promoting glycolysis and resulting in increased lactate accumulation. In physiological conditions, MCT1 to MCT4 operate in concert to maintain the cell-to-cell lactate balance. However, under anaerobic conditions within the tumor microenvironment, cancer cells rely on lactate produced by glycolysis as an energy source, a process known as “metabolic symbiosis”, and require MCT1 and MCT4 for the transportation of lactate. Therefore, the targeted regulation of these proteins to inhibit lactate production is of great significance for disease treatment. In the following section we summarize the effects of different targeted small molecule drugs on lactate, as shown in Table 2.

### 7.1. Targeted Small Molecule Inhibitors for HK-2

HK-2, the initial rate-limiting enzyme in glycolysis, is a significant lactate producer [4] and is overexpressed in numerous diseases [111]. Since 2-deoxy-D-glucose (2DG), a glucose competitor, was designed in 1950 [112], it has shown anti-tumor activity in various cancers [113,114,115,116,117] by inhibiting glycolysis and ATP synthesis, among other mechanisms [118]. However, its clinical development was halted due to adverse effects [119]. Recent discoveries have unveiled novel HK-2 inhibitors with potential therapeutic implications. Benzenserazide (Benz) specifically targets HK-2, reducing glucose uptake and lactate production and potentially triggering apoptosis in rectal cancer cells [120]. The pyruvate analog 3-bromopropanoic acid (3-BP) inhibits HK-2, curbing colorectal cancer cell proliferation through ferroptosis, autophagy, and apoptosis [121]. Metformin [122] and Pachymic [123] acid emulate the effects of glucose-6-phosphate (G6P) to suppress HK-2 expression, thereby inhibiting its proliferation in hepatocellular and breast cancer cells. Ikarugamycin also inhibits HK-2, reducing glycolysis and lactate production in pancreatic cancer cells [124]. VDAC, which is crucial for mitochondrial metabolite transport [125], favors ATP transfer from mitochondria to cytoplasm via VDAC1 on the OMM, enhancing glycolysis [126]. The inhibition of HK2-VDAC1 binding by Chrysin [127] and Piperlongumine [128] prevents glycolysis and lactate production, exhibiting anti-tumor effects in liver and lung cancer cells. Additionally, (-)-Epigallocatechin Gallate (EGCG) protects against renal injury by mitigating mitochondrial dysfunction and Drp1-induced fission [129]. In acute kidney injury (AKI), the increased HK-2 expression and lactate production further upregulate HK-2, with AST-120 being shown to ameliorate AKI via downregulating HK-2 and reducing H3K18 lactylation [130]. 

HK-2 inhibitors are categorized into three classes: glucose-competitive, G6P analog, and HK2-VDAC1-binding inhibitors. These collectively reduce lactate production by inhibiting HK-2, offering a disease-modifying approach.

### 7.2. Targeted Small Molecule Inhibitors for LDHA

LDHA, a catalyst in lactate synthesis, is upregulated in numerous pathologies, such as tumors [131], liver fibrosis [132], pulmonary fibrosis [133], sepsis [134], and hypertension [135]. The pursuit of potent and selective LDHA inhibitors is a significant therapeutic strategy for these conditions. Oxamate, resembling pyruvate, competes with it to inhibit LDHA [136], impeding the growth of aggressive tumors like pituitary adenomas, medulloblastoma, and glioblastoma by modulating the glycolytic pathway [137]. Hydroxyisoxazole-4-carboxylic acid (HICA) [138] and 1-(phenylseleno)-4-(trifluoromethyl) benzene (PSTMB) [139] also act as pyruvate competitors, inhibiting LDHA and disrupting mitochondrial membrane integrity, thereby inhibiting the proliferation of human colon cancer cells. The NAD+/NADH balance is crucial to lactate synthesis [140], with LDHA reducing pyruvate to lactate and NAD+ to NADH [141]. Gossypol [142] and FX-11 [143], which compete with NADH, exhibit anti-fibrotic effects and promote lymphoma cell death. Galloflavin, an NADH competitor, inhibits LDHA isoforms, disrupting aerobic glycolysis and reducing the viability of various cancer cells [144]. LDHA-IN3 [145] and Azm-33 [146] are LDHA inhibitors with unclear mechanisms, which have been shown to inhibit melanoma proliferation and promote cell death in breast and colon cancers, respectively. GPEG-140, an inhibitor of LDHA, reduces lactate production and histone lactylation, exerting anti-pulmonary fibrosis effects [147]. Celastrol, a natural anti-inflammatory compound, binds to LDHA and mitigates sepsis-induced damage [148]. Eriocitrin (ERI) [149], a flavonoid, reduces lung inflammation by inhibiting LDHA and glycolysis in a mouse model of acute lung injury. Suberoylanilide hydroxamic acid (SAHA) increases LDHA acetylation, reducing its activity and glycolysis rate and alleviating sepsis [150]. 

LDHA inhibitors are categorized into three classes: pyruvate competitors, NADH competitors, and other inhibitors. These collectively inhibit LDHA, reducing lactate production and showing therapeutic potential for various diseases, particularly in oncology. Accordingly, LDHA inhibitors are emerging therapeutic targets. 

### 7.3. Targeted Small Molecule Inhibitors for HIF-1α

In hypoxic conditions, HIF-1α activates glycolytic genes like HK-2, enhancing lactate production [151], a process that is often upregulated in cancer [152]. Targeting HIF-1α to suppress glycolysis is a therapeutic strategy, with compounds like PX-478 showing promise in enhancing mitochondrial function, reducing lactate, and improving pancreatic β cell function in diabetes in clinical trials [153,154]. Oligomycin inhibits ATP synthesis by blocking H+-ATP synthase, indirectly affecting HIF-1α stability. It also reduces lactate production in senescent cells by downregulating HIF-1α [155,156]. Salvianolic acid A (SAA) treats cardiac fibrosis by inhibiting HIF-1α-driven glycolysis through the Akt/GSK-3β pathway [157]. ω-alkynyl arachidonic acid [158] and Steppogenin [159] modulate HIF-1α to promote anti-inflammatory responses and exhibit anti-tumor effects. Albendazole down regulates HIF-1α in non-small cell lung cancer (NSCLC) cells, inhibiting glycolytic enzymes and lactate formation and thereby curbing cancer proliferation [160]. CRLX101 [161], a nanoparticle-drug conjugate, delivers camptothecin to cancer cells, sustaining HIF-1α inhibition and showing synergistic effects with anti-angiogenesis drugs like bevacizumab in rectal cancer models. SENP-1, a SUMOylation-specific protease, stabilizes HIF-1α by removing SUMO modifications, influencing its activity under hypoxia [162,163]. Chloramphenicol promotes autophagy and disrupts the HIF-1α/SENP-1 interaction, inhibiting glycolysis and the proliferation of NSCLC [164,165]. HIF-1α inhibitors can be direct, targeting HIF-1α activity, or indirect, modulating functions like H+-ATP synthase or HIF-1α-SENP-1 interactions. Understanding these mechanisms is key to developing effective HIF-1α inhibitors for cancer and other diseases.

### 7.4. Targeted Small Molecule Inhibitors for MCT1 or MCT4

Monocarboxylate transporters MCT1 and MCT4 play a significant role in the metabolism of lactate. MCT1 and MCT4 have been proposed as potential therapeutic targets in a variety of disease treatment strategies [166,167,168]. As a pyrimidine derivative, AR-C155858 exhibits high specificity for MCT1. In a mouse breast cancer model, AR-C155858 demonstrated efficacy in inhibiting extracellular lactate uptake and exerted an inhibitory effect on tumor growth [169]. AZD3965, a derivative of AR-C155858, is currently undergoing phase I clinical trials. Azd3965 is a selective MCT1 inhibitor with a Ki value of 1.6 nM. Studies have shown that it can impede the growth and proliferation of breast cancer cells by inhibiting MCT1 activity [170]. AZD0095 is a clinical candidate for MCT4, developed by AstraZeneca. Obtained through a high-throughput phenotypic screening approach, the resulting compound, AZD0095, is optimized through multiple rounds of synthesis and has been shown to be highly active and highly MCT1-selective. Pharmacological studies have demonstrated that the compound affects the transport of lactate in lung cancer cells, ultimately leading to the inhibition of their growth [171].

**Table 2:** Small molecule drugs, their inhibitory mechanisms, and their roles in diseases or cells: an overview of the most significant lactate drugs.

Small Molecules Drugs	Mechanism	Function	Refs.
**Targeted inhibition of HK-2 to decrease lactate**			
2-DG	Competition with glucose	Against a variety of solid tumors	[112,113,114,115,116,117,118,119]
Benz	Specific binding to HK2	Induction of apoptosis in rectal cancer cells	[120]
3-BP	Pyruvate acid analogs	Induction of ferroptosis, autophagy and apoptosis to inhibit the proliferation of colorectal cancer cells	[121]
Metformi	Mimics the physiological effects of G6P	Inhibits the proliferation of hepatocellular carcinoma	[122]
Pachymic acid		Inhibition of breast cancer cell proliferation	[123]
Ikarugamycin		Inhibits the proliferation of pancreatic cancer cells	[124]
Chrysin	Inhibition of the binding between HK2 and VDAC	Inhibits the proliferation of hepatocellular carcinoma	[125]
Piperlongumine		Inhibits the proliferation of non-small cell lung cancer cells	[128]
EGCG	Inhibition of mitochondrial fission	Alleviates kidney damage	[129]
AST-120	Direct inhibition of HK-2	Alleviates acute kidney injury	[130]
**Targeted inhibition of LDHA to decrease lactate**			
Oxamate	Structurally similar to pyruvate	Against a variety of solid tumors	[136,137]
HICA		Destroys the integrity of mitochondrial membrane and inhibits the proliferation of human colon cancer cells	[138]
PSTMB			[139]
Gossypol	Compete with NADH	Alleviates pulmonary fibrosis	[142]
FX-11		Promotes lymphoma cell death	[143]
Galloflavin		Inhibits the proliferation of various cancer cells	[144]
LDHA-IN3	Unknown mechanism of action	Inhibits the proliferation of melanoma cells	[145]
Azm-33		Inhibits the proliferation of MCF-7 and HCT116	[146]
GPEG-140		Alleviates pulmonary fibrosis	[147]
Celastrol	Direct inhibition of LDHA	Alleviates sepsis	[148]
ERI		Alleviates acute lung injury	[149]
SAHA	Promotion of LDHA acetylation	Alleviates sepsis	[150]
**Targeted inhibition of HIF-1α to decrease lactate**			
PX-478	Directly inhibits HIF-1α	Prevents gastric mucosal lesions	[153]
		Improves diabetes	[154]
Oligomycin	Inhibiting the enzyme H+-ATP synthase	Inhibition of senescent cells	[155]
SAA	Inhibiting HIF-1α	Alleviates myocardial fibrosis	[157]
ω-alkynyl arachidonic acid	HIF-1α was induced to bind to the HRE sequence in the iNOS promoter	Improvement in myocardial infarction	[158]
Steppogenin	Directly inhibits HIF-1α	Inhibits the proliferation of HEK293T cells	[159]
Albendazole	Directly inhibits HIF-1α	Anti-NSCLC	[160]
CRLX101	Directly inhibits HIF-1α	Synergizes with Avastin against rectal cancer	[161]
Chloramphenicol	Inhibition of the HIF-1α/SENP-1 protein interaction	Promotes autophagy in non-small cell lung cancer cells	[164]
**Targeted inhibition of MCT1 or MCT4 to decrease lactate**			
AR-C155858	Inhibition of MCT1	Anti-breast cancer	[169]
AZD3965		Inhibits the proliferation of breast cancer cells	[170]
AZD0095	Inhibition of MCT4	Inhibits the proliferation of lung cancer cells	[171]

## 8. Conclusions and Perspectives

In vivo, cells consume energy to decompose and synthesize molecules. Glucose, a key molecule for energy supply, is converted to pyruvate via the glycolytic process, and pyruvate produced by this process is often regarded as the core of the cellular energy metabolism. In healthy cells, pyruvate is transported into the mitochondria, where it undergoes oxidative phosphorylation to release energy. Conversely, under conditions of hypoxia or during high-intensity exercise, cells rely on glycolysis to produce lactate as a rapid source of energy [17,53]. Notably, the excessive accumulation of lactate has been closely associated with the pathogenesis and progression of various diseases, including liver disorders, tumors, and cardiovascular diseases [17,74,77,83]. Therefore, a comprehensive understanding of lactate metabolism and its implications in disease processes is crucial for developing novel therapeutic strategies. lactate was once regarded as a simple by-product of glycolysis. With further research, its important physiological and pathological functions in organisms were gradually revealed. Histone lactylation modification, proposed in 2019, further amplified the importance of lactate [2], and its roles in the cellular energy supply [17], signal pathway regulation [35], epigenetic modification [2], and fat metabolism regulation [40] were gradually revealed. Lactate is not only an energy-provider to cells under hypoxic conditions; it is also a signaling molecule that can regulate the physiological functions of cells. When oxygen is in short supply, cells produce lactate through glycolysis as an alternative energy source to power the cells [53]. At the same time, lactate can also regulate cellular fat metabolic activity by affecting the synthesis of intracellular fatty acids [165]. Even more exciting is the new role of lactate in epigenetics. The discovery of histone lactylation modification has opened a new perspective for us to understand the role of lactate in the regulation of gene expression [2]. This modification may have similar functions to acetylation, methylation, and acetic acid modifications, and affect gene transcription and expression by changing the structure and stability of histones [27]. Although the study of gene expression changes after histone lactylation modification is still in its infancy, this field undoubtedly has great research potential and application value. However, lactate accumulation is a problem that cannot be ignored in many diseases. Although lactate is often used as an important indicator for the diagnosis of certain diseases, studies have also found that supplementing blood vessels with lactate can alleviate heart disease [44,45,46,47,48]. In tumors, the accumulation of lactate not only provides energy for tumor cells but also participates in the regulation of immune responses and angiogenesis in the tumor microenvironment, promoting tumor growth and metastasis [54,55]. In liver fibrosis [74], sepsis [77], ischemic stroke [83], myocardial infarction [95], and acute kidney injury [102], the changes in lactate levels are also closely related to the severity and prognosis of the disease. These findings suggest that lactate may become an important target for the diagnosis and treatment of disease. When targeting the regulation of lactate, several small molecule inhibitors have shown therapeutic potential. Lactate production and transport are affected by key enzymes such as HK-2 [4] and LDHA [126], as well as MCT1 and MCT4 transporters [166,167,168]. Through the regulation of these key proteins, the production and transport of lactate can be regulated; then, the process of the disease can be alleviated. Inhibitors of HK-2 are broadly divided intoglucose-competitive inhibitors (e.g., 2-DG) [112], direct inhibitors (e.g., Benz [120], 3-BP [121]), and inhibitors that mimic the physiological effects of glucose-6-phosphate (G6P) (e.g., Metformin [122] and Pachymic acid [123]). In addition, both Chrysin [127] and piperlongumine [128] inhibited glycolysis by inhibiting the binding of HK2 to VDAC1, which in turn reduced lactate production. LDHA inhibitors are mainly divided into pyruvate-competitive inhibitors, coenzyme (NADH)-competitive inhibitors, and other mode inhibitors. For example, Oxamate [137], as a pyruvate-competitive inhibitor, was shown in preclinical studies to regulate the glycolytic pathway and block tumor growth by specifically inhibiting LDHA. Gossypol [142] and FX-11 [143], as coenzyme-competitive inhibitors, also showed anti-disease effects. In addition, there are other modalities of inhibitors, such as LDHA-IN3 [145] and Azm-33 [146], which inhibit cell proliferation by inhibiting LDHA in melanoma and breast cancer cells. Inhibitors targeting HIF-1α, such as PX-478 [153], have shown therapeutic potential by reducing the activity of HIF-1α to reduce lactate production. In addition, Oligomycin [156], Steppogenin [149], and Albendazole [160] also reduce lactate production by inhibiting HIF-1α. CRLX101 [161], a novel nanomedicine, shows synergistic effects with antiangiogenic drugs by directly inhibiting HIF-1α. These findings suggest that glycolysis and lactate production can be effectively inhibited by targeting HIF-1α and its related pathways, providing new strategies for the treatment of cancer and other diseases. By regulating the MCT1 and MCT4 transporters, the transport of lactate between different cells can be further regulated, thus having a regulatory effect on the disease. AR-C155858 [169] and AZD3965 [170], as pyrimidine derivatives, have been shown to inhibit MCT1 with high specificity. In addition, AZD0095 [171], as an MCT4 inhibitor, also affected lactate transport. Future research may focus on the following aspects: the discovery of novel therapeutic targets, the search, and validation of new therapeutic targets based on new findings in lactate metabolism and signaling, and the development of clinical treatment strategies, including drug development, gene therapy, immunotherapy, and other novel treatment strategies. Future studies also need to focus on the interaction between lactate and other metabolites, such as ketone bodies, and fatty acids, and their common mechanisms of action in disease. The biological functions of lactate are far more complex than previously appreciated, and a deep understanding of the metabolic and signaling roles of lactate will provide new strategies and targets for the diagnosis and treatment of diseases. Future studies will reveal the full role of lactate in health and disease and provide more options for clinical treatment.

## Data Availability

All data are contained within the article.
